# Hidden Drug Rash With Eosinophilia and Systemic Symptoms (DRESS): A Rare Case of Preceding Organ Damage Before Skin Manifestations

**DOI:** 10.1002/ccr3.72001

**Published:** 2026-02-23

**Authors:** Kent Sato, Satoshi Kawaguchi, Shunsuke Nakajima, Akihito Tampo, Motoi Okada

**Affiliations:** ^1^ Department of Emergency Medicine Asahikawa Medical University Asahikawa Japan

**Keywords:** acute kidney injury, allergy, DIHS, DRESS, drug‐induced hypersensitivity syndrome, minocycline

## Abstract

Drug rash with eosinophilia and systemic symptoms (DRESS) is a drug‐induced hypersensitivity reaction that can cause significant organ injury. Unlike anaphylaxis, it often goes unnoticed because the skin eruptions develop slowly. We report the case of a 75‐year‐old woman who presented with eosinophilia and acute kidney injury. Two weeks earlier, she had been treated with minocycline hydrochloride for a giant liver cyst. No rash was initially observed, but by the third day of admission, a widespread eruption appeared. She was diagnosed with minocycline‐induced DRESS and treated with oral prednisolone, which was tapered after hemodialysis was discontinued. She was later transferred back to her previous hospital for rehabilitation. This case highlights that many drugs can trigger DRESS syndrome and that serious organ damage may precede the onset of rash. Clinicians should remain alert to this possibility when evaluating patients with unexplained organ dysfunction.

## Key Clinical Message

DRESS is a severe drug reaction in which organ injury may appear before the rash. A 75‐year‐old woman developed eosinophilia and kidney injury after minocycline, later showing a full‐body rash and improving with steroids. The case highlights the need for early recognition of DRESS.

## Graphical abstract

In DRESS syndrome, severe organ damage can occur after drug administration, sometimes even before the appearance of a rash. Clinicians should keep this possibility in mind when evaluating organ dysfunction of unknown cause.

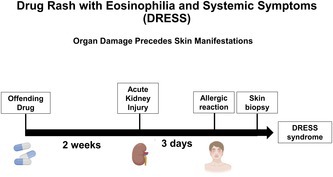

## 1 Introduction

Drug rash with eosinophilia and systemic symptoms (DRESS) is a drug‐induced hypersensitivity syndrome involving skin rashes, hematologic abnormalities, lymphadenopathy, and internal organ damage leading to death.

DRESS and drug‐induced hypersensitivity syndrome (DIHS) are similar conditions, except that the diagnostic criteria for DRESS do not focus on herpesvirus 6 infection [1].

Although regarded as the same clinical entity as DIHS, DRESS includes a wider range of drug eruptions.

The incidence of DRESS is estimated to be 1 case per 10,000–100,000 individuals. Many patients remain undiagnosed because of the infrequency of DRESS and the difficulties involved in its diagnosis [2]. In this report, we present an unusual case of DRESS developing into severe acute kidney injury (AKI) before manifesting as a skin rash.

## 2 Case History/Examination

A 75‐year‐old woman was transferred to our hospital for urgent management of AKI. Her medical history included hypertension and dyslipidemia but no allergies. Three weeks ago, in another hospital, she had received minocycline hydrochloride injection therapy for a giant liver cyst. However, her serum creatinine levels rose sharply 2 weeks after the injection, and general malaise with high fever was observed.

Upon admission to our hospital, the patient's vital signs were as follows: blood pressure, 153/92 mmHg; heart rate, 89 beats/min; respiratory rate, 20 breaths/min; percutaneous oxygen saturation, 96%; and body temperature, 38.5°C. Careful physical examination by multiple doctors revealed no obvious abnormal findings, including skin rashes. Blood tests revealed a white blood cell count of 24.3 × 10^4^/μL and an eosinophil count of 2.18 × 10^3^/μL (Table 1). Creatinine level was 6.46 mg/dL, and C‐reactive protein level had increased to 15.19 mg/dL. Tests for antinuclear antibodies; hepatitis A, B, and C viruses; and other viruses returned negative results.

**TABLE 1:** Laboratory data of the patient upon admission.

Laboratory tests	Results	Normal range
White blood cell (/μL)	24,300	3300–8600
Neutrophil (/μL)	20,900	1500–7500
Eosinophil (/μL)	2180	0–500
Lymphocyte (/μL)	730	100–400
Hemoglobin (g/dL)	11.2	11.6–14.8
Platelet (×10^3^)	332	158–348
Prothtombin time (% in the normal range)	61	70–131
Activated partial thromboplastin time (s)	41.4	27.0–39.9
D‐dimer (μg/mL)	9.9	0–0.50
Total protein (g/dL)	5	6.6–8.1
Albmin (g/dL)	1.8	4.1–5.1
Total bilitubin (mg/dL)	0.3	0.4–1.5
Amylase (mg/dL)	40	44–132
Alkaline phosphatasec (U/L)	100	38–113
Aspartate aminotransferase (U/L)	23	13–30
Alanine aminotransferase (U/L)	38	7–23
γ‐glutamyl transpeptidase (U/L)	47	9–32
Creatinine (mg/dL)	6.46	0.46–0.79
Blood urea nitrogen (mg/dL)	45.4	8.0–20.0
C‐reactive protein (U/L)	15.19	≦ 0.14

*Note:* Laboratory data demonstrated increases in the counts of white blood cells and eosinophils as well as in the level of C‐reactive protein. In addition, the levels of blood urea nitrogen and creatinine indicated severe renal failure.

**FIGURE 1:**
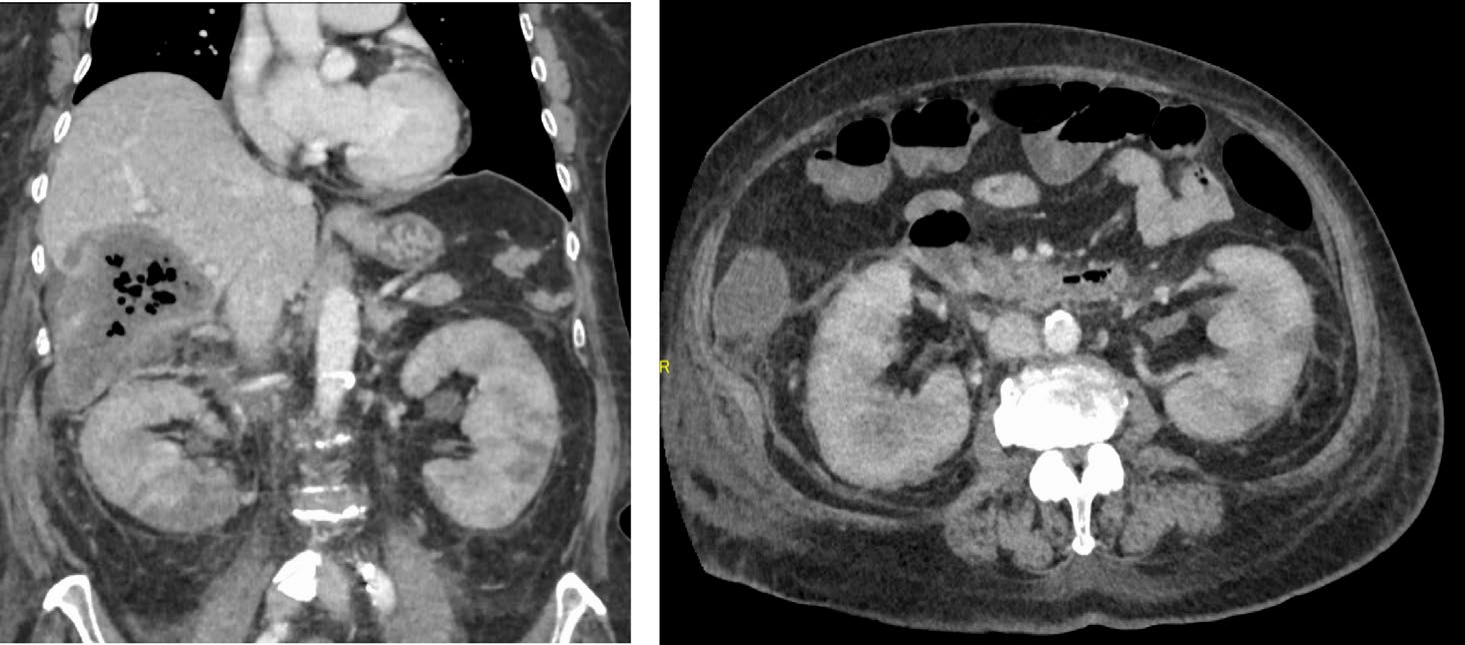
Abdominal enhanced computed tomography. A contrast computed tomography scan demonstrated a giant liver cyst and bilateral renal enlargement with poor contrast area.

A contrast computed tomography scan highlighted a giant liver cyst and bilateral renal enlargement with regions of poor contrast (Figure 1).

## 3 Differential Diagnosis, Investigations, and Treatment

**FIGURE 2:**
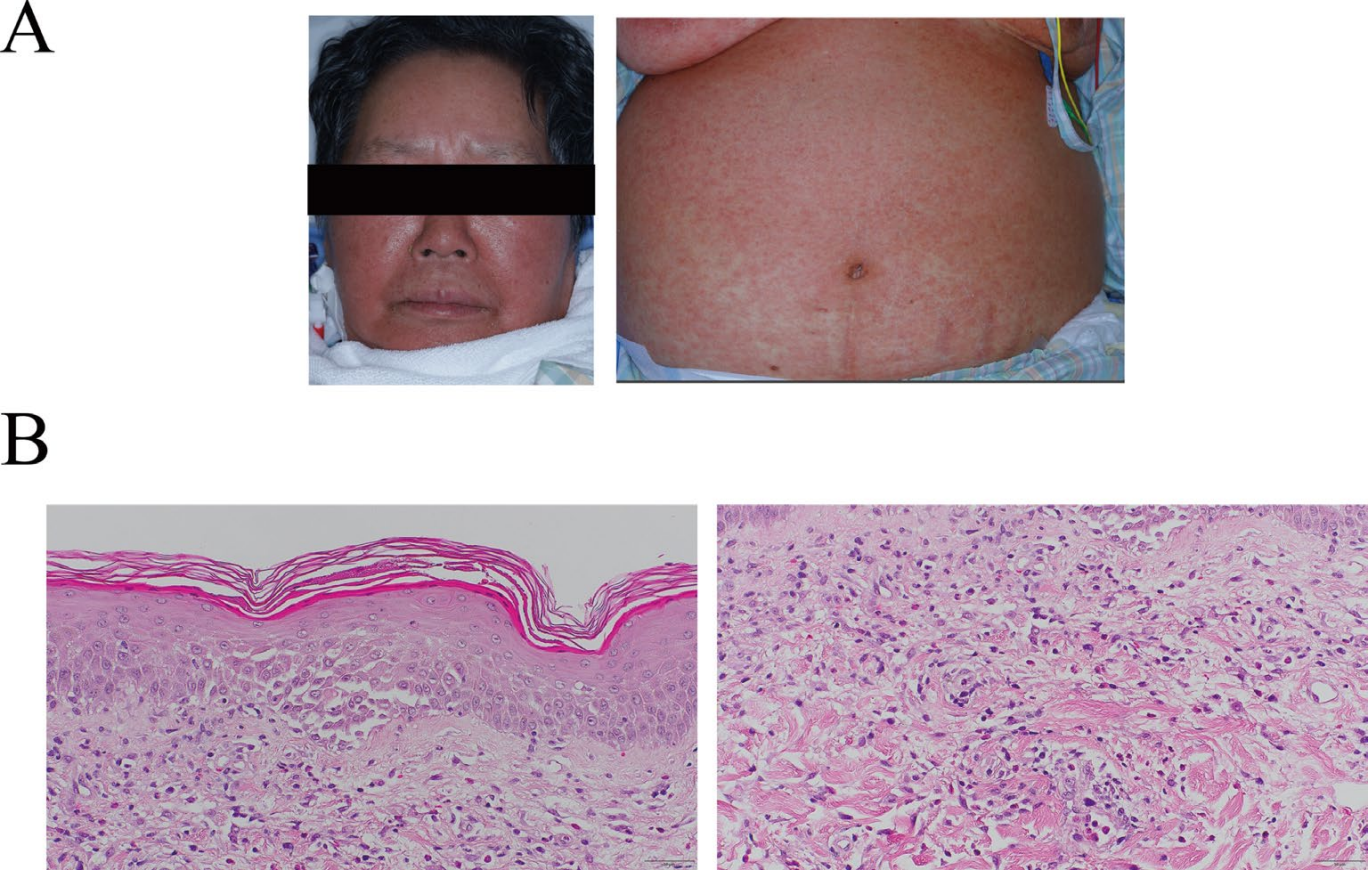
(A) Rash on patient's face and body. On the third day of admission, the patient suddenly developed edematous erythema on her face, body, and limbs, which eventually covered > 70% of her body. These findings were not observed prior to the onset of acute kidney injury. (B) Pathological findings from abdominal skin biopsy. Skin biopsy of the abdominal lesion showed basal cell vacuolar degeneration, infiltration of lymphocytes in the upper dermis, and scattered eosinophils and neutrophils in the dermal stroma.

Initially, we suspected a drug or sepsis as the cause of AKI. Considering the possibility of urinary infection, we administered meropenem and initiated hemodialysis as a treatment for AKI. On the third day of hospitalization, a rash suddenly appeared on the face, trunk, and extremities. The rash, considered as edematous erythema, progressed to cover > 70% of the body surface area (Figure 2A).

Considering the possibility of a dialysis‐related allergy, we changed the dialysis membrane and dialysis fluid, but her allergic condition did not improve. Drug‐induced lymphocyte stimulation tests for the dialysis fluid, dialysis membrane, meropenem, minocycline, and other antibiotics also returned negative results. A blood test for herpesvirus 6 infection yielded negative results. On the eighth day of hospitalization, bone marrow biopsy and skin biopsy were performed but renal biopsy was not because of refusal from the patient. The bone marrow biopsy was not indicative of hematological malignancy; however, the abdominal skin biopsy revealed infiltration of lymphocytes and eosinophils into the upper dermis, which is consistent with the pathophysiology of severe drug reaction (Figure 2B).

On the basis of these results and the clinical course, we suspected DRESS caused by minocycline. The diagnosis of DRESS was confirmed after calculating the European Registry of Severe Cutaneous Adverse Reactions score, which was 6 out of 9 points: 2 for increased eosinophils, 1 for extensive skin rash, 1 for rash suggestive of DRESS, 1 for renal involvement, and 1 for exclusion of other causes.

**FIGURE 3:**
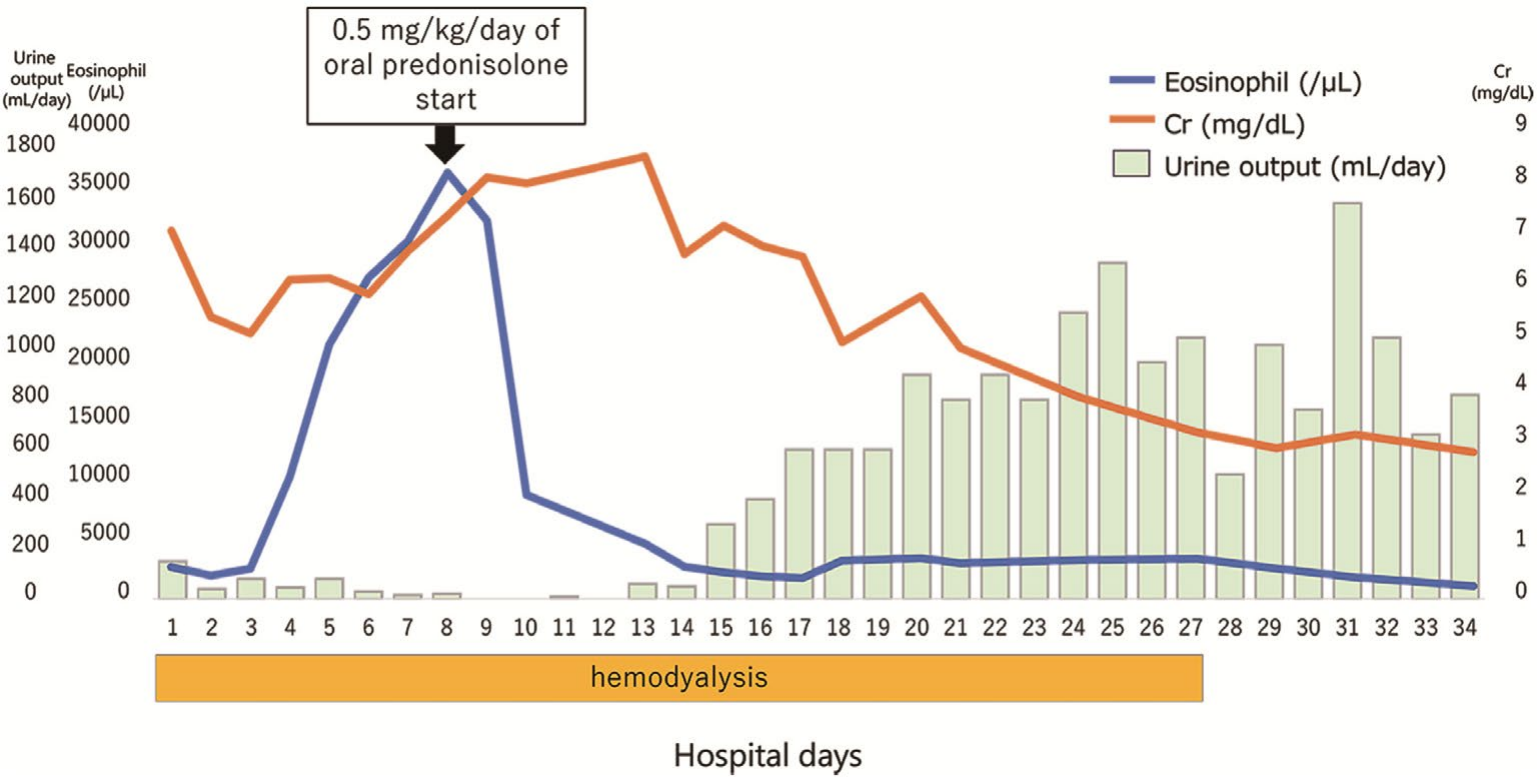
Time series of creatinine level, urine volume, and eosinophil count. Upon admission, the patient experienced oliguric renal failure, and eosinophil count and creatine level increased day by day. On the eighth day of admission, we administered prednisolone at a dose of 0.5 mg/kg/day, which dramatically improved the patient's condition and renal failure, while lowering her creatine level and eosinophil count. Finally, hemodialysis was stopped, and the patient was transferred to her previous hospital for rehabilitation on the 34th day.

We administered prednisolone at a dosage of 0.5 mg/kg/day, which dramatically improved the systemic response, renal failure, and eosinophilia (Figure 3). In addition, because reactivation of cytomegalovirus was confirmed through blood tests, we started the administration of ganciclovir on the 16th day of hospitalization. The patient eventually no longer required hemodialysis. The dosage of prednisolone was tapered, and subsequently it was discontinued. On the 34th day of hospitalization, the patient was transferred to the previous hospital for rehabilitation. Regular monitoring at the previous hospital has confirmed that the patient did not develop late autoimmune sequelae after discontinuation of prednisolone.

## 4 Conclusion and Results

We report a case of minocycline‐induced DRESS that developed in a patient with AKI and eosinophilia. DRESS is a drug‐induced hypersensitivity syndrome involving skin rashes, hematological abnormalities, lymphadenopathy, and internal organ damage leading to death. The early diagnosis of DRESS is challenging because of the delayed manifestation of symptoms and low awareness.

Clinicians should remember that commonly used drugs can trigger DRESS. Moreover, DRESS can manifest in severe internal organ dysfunction before the appearance of any physical allergic reaction.

## 5 Discussion

Here, we report a case of minocycline‐induced DRESS involving AKI. DRESS involves a drug‐induced hypersensitivity reaction characterized by the slow onset of a drug rash from drug exposure, diverse clinical manifestations, and a prolonged clinical course. Alarmingly, in some cases, DRESS can cause irreversible organ dysfunction and even death. In this case, treatment was delayed because of the absence of cutaneous manifestations despite the onset of AKI.

Three points should be considered from this case. First, many commonly used drugs could cause DRESS. Indeed, previous reports of DRESS have included aromatic anticonvulsants, sulfonamides, salazosulfapyridines, allopurinol, calcium channel blockers, and terbinafine [2, 3]. Moreover, the long latency period makes the diagnosis of DRESS challenging. Minocycline is also a well‐known drug that can cause DRESS [3, 4, 5]. DRESS typically occurs > 2 weeks after the start of administration of the causative drug [2]. The risk of DRESS may be higher in patients with chronic kidney disease, given that minocycline is primarily excreted by the kidneys.

Second, DRESS can sometimes lead to internal organ dysfunction, which may manifest as hepatitis, nephrotoxicity, pneumonitis, cerebral edema, eosinophilia, pericardial effusion, leukocytosis, myocarditis, or thyroiditis [5, 6]. Patients with DRESS are estimated to have a mortality rate of 10%–20%, and most of the cases resulting in death involve myocarditis or hepatotoxicity. Recent systematic reviews have highlighted that antibiotics are the most common cause of death in women with kidney or liver lesions.

In the present case, the patient required hemodialysis owing to AKI. According to previous estimates, 15%–35% of patients with DRESS present with AKI, and 30% of patients with AKI require temporary renal replacement therapy [7, 8]. In addition, DRESS is known to cause acute interstitial nephritis regardless of the presence of tubular necrosis [9].

The mortality rate of patients with AKI associated with DRESS is 13% [6, 7, 8]. Moreover, case reports have described young patients developing acute renal failure due to lamotrigine‐induced DRESS [10]. Therefore, early diagnosis and treatment of this disease are critical.

DRESS is associated with the risk of cytomegalovirus reactivation [11], which can cause complications such as gastrointestinal bleeding, hepatitis, and myocarditis [12].

Third, organ damage can sometimes precede general allergic reactions, such as skin rashes, bronchial asthma, and systemic edema. The patient in this study initially presented with AKI, increased eosinophils, and fever; a generalized skin rash appeared > 1 week after the onset of AKI. The European Registry of Severe Cutaneous Adverse Reactions scoring system for DRESS diagnosis relies on the presence of cutaneous symptoms. Therefore, unexplained eosinophilia, unexplained fever, and organ dysfunction preceding cutaneous symptoms may result in misdiagnosis and delay in treatment.

Genetic polymorphisms of metabolic enzymes are sometimes screened for in patients starting antiepileptic drug therapy, but this does not necessarily confirm a diagnosis [13].

Long‐term follow‐up is also necessary because up to 25% of patients with DRESS experience recurrence or develop late‐onset autoimmune sequelae, such as thyroiditis, vitiligo, type 1 diabetes, and systemic lupus erythematosus.

Therefore, DRESS should be suspected on the basis of not only dermatological findings or eosinophilia but also systemic symptoms, including organ dysfunction. More importantly, DRESS need not necessarily precede skin symptoms, so close attention must be paid to the patient's medical history and physical examination over time.

## Author Contributions

**Kent Sato:** conceptualization, data curation, investigation, validation, visualization, writing – original draft. **Satoshi Kawaguchi:** conceptualization, investigation, writing – original draft, writing – review and editing. **Shunsuke Nakajima:** conceptualization, investigation. **Akihito Tampo:** conceptualization, investigation, supervision. **Motoi Okada:** conceptualization, formal analysis, funding acquisition, investigation, project administration, supervision, writing – original draft, writing – review and editing.

## Funding

The authors have nothing to report.

## Consent

K.S. obtained written informed consent from the patient to publish her details.

## Conflicts of Interest

The authors declare no conflicts of interest.

## Data Availability

Data available on request due to privacy/ethical restrictions. The data that support the findings of this study are available on request from the corresponding author. The data are not publicly available due to privacy or ethical restrictions.
